# Mystery Disease X Outbreak in the Democratic Republic of the Congo: A Narrative Review of Epidemiological Patterns and Response Challenges

**DOI:** 10.1002/hsr2.72263

**Published:** 2026-04-02

**Authors:** Muhammad Shaheer Bin Faheem, Muhammad Haroon‐Ul‐Rasheed, Sumaya Samadi

**Affiliations:** ^1^ Department of Medicine and Surgery Karachi Institute of Medical Sciences, KIMS Karachi Sindh Pakistan; ^2^ CMH Multan Institute of Medical Sciences, CIMS Multan Punjab Pakistan; ^3^ Kabul University of Medical Sciences “Abu Ali Ibn Sina” Kabul Afghanistan

**Keywords:** Democratic Republic of Congo, epidemic, future strategies, infectious disease, World Health Organization

## Abstract

**Background and Aims:**

An unknown disease known as Disease X first surfaced in the Panzi Health Zone, Kwango Province, Democratic Republic of the Congo (DRC), towards the end of 2024. It mainly affected children under the age of five. The clinical characteristics and of the outbreak are examined in this review.

**Methods:**

A narrative review was conducted by searching PubMed, WHO reports, and gray literature regarding this outbreak before 2025.

**Results:**

The epidemic manifests as flu‐like symptoms, fever, and cough. Response to fighting this outbreak has been hindered by socioeconomic problems, limited testing, and poor health facilities. The outbreak reflects similar problems from previous outbreaks, such as Ebola and cholera.

**Conclusion:**

The Disease X outbreak highlights problems in the DRC's health system. Strengthening testing facilities, funding, and broad surveillance is essential in controlling this outbreak.

## 1 Introduction

A notification about a sudden upsurge in mortality in the Panzi health zone from an unidentified cause was sent to WHO by the Democratic Republic of Congo's (DRC) Ministry of Public Health on November 29, 2024. A total of 406 instances of an unidentified illness were reported between October 24 and December 5, 2024, including 31 fatalities [1]. A sharp rise in the number of incidents that fit the criteria of this condition; as of December 16, 891 cases have been reported [2]. Apollinaire Yumba, the provincial minister of health, and Remy Saki, the deputy governor of Kwango province, reported on Monday that the affected individuals experienced flu‐like symptoms and headaches. To detect the illness, a medical team was dispatched to the Panzi health zone to gather samples and conduct an analysis [3]. Being an underdeveloped country, the DRC faces numerous challenges, including a shortage of medical facilities and labs that make it hard to properly diagnose and treat emerging medical emergencies [4]. This new disease outbreak continues to grow and needs prompt action to address the ongoing situation [1].

This review aims to investigate the disease outbreak in detail, concentrating on its salient features, symptoms, and the difficulties in dealing with it. It also looks into vulnerable communities affected by this disaster by identifying diagnostic gaps and providing helpful suggestions.

## 2 Methodology

Current information regarding the Disease X outbreak in the Panzi Health Zone, Kwango Province, DRC, is summarized in this narrative review. Databases including PubMed, Google Scholar, and WHO reports were used; the search was carried out with an emphasis on research published before 2025. “Disease X,” “infectious disease,” “Democratic Republic of Congo,” “epidemics,” were among the keywords used. Field reports and pertinent gray literature were also examined. The results were examined to draw attention to prospective response tactics, healthcare issues, and epidemiological patterns.

## 3 Background: Health Landscape

The DRC people generally have a poor health status. Serious infectious conditions like monkeypox, cholera, meningitis, plague, poliomyelitis, measles, and hemorrhagic fever caused by viruses pose great epidemic risks. Other infections like human African trypanosomiasis, tuberculosis, and malaria are prevalent across the country at large. HIV also impacts around 4.5% of people between the ages of 15 and 49 [5]. Even the World Health Organization (WHO) views DRC as a country with a “high burden” for both HIV and tuberculosis cases [6]. People in the poor regions of the country, both male and female, are of the opinion that the government should be more accountable and considerate of health issues. Concerns were also brought up about a lack of confidence in the resources, abilities, knowledge, and motives of healthcare professionals and practitioners connected to the healthcare sector [7].

## 4 Epidemiology of the Current Outbreak

According to the local authorities, the disease outbreak is mainly in the southwest of DRC and has killed around 143 individuals in November alone [3]. From October to December, 406 cases were reported in the Kwango province of DRC. Nine of the Panzi health zone's 30 health areas—Kambandambi, Kanzangi, Kasanji, Mwini ngulu, and Tsakala Panzi Kahumbulu, Kiama, Mbanza Kipungu, Makitapanzi—have recorded cases. The health regions of Tsakala Panzi, Makitapanzi, and Kanzangi report the bulk of cases. Tsakala Panzi had 169, Makitapanzi had 142, and Kanzangi had 78 reported incidences respectively [1]. As of December 16, testing results from 430 specimens showed positive tests for human corona and some other viruses, malaria, and common respiratory viruses (Influenza A (H1N1, etc.). Even though more laboratory testing is still being done, these results collectively imply that a mix of falciparum malaria, common and seasonal viral respiratory infections, and acute malnutrition caused a rise in severe diseases and fatalities that disproportionately affected children under five [2]. Blood, oropharyngeal and nasopharyngeal swabs, urine, and breastmilk specimens were among the 430 samples that were taken from probable cases in the Panzi health zone. 55 (62%) of the 88 quick diagnostic testing for malaria that were conducted were positive. Furthermore, 17 (65%) of the 26 samples examined using the PCR test which checks for distinct pathogens, including several viral hemorrhagic fevers—tested positive for Plasmodium falciparum [2].

**FIGURE 1:**
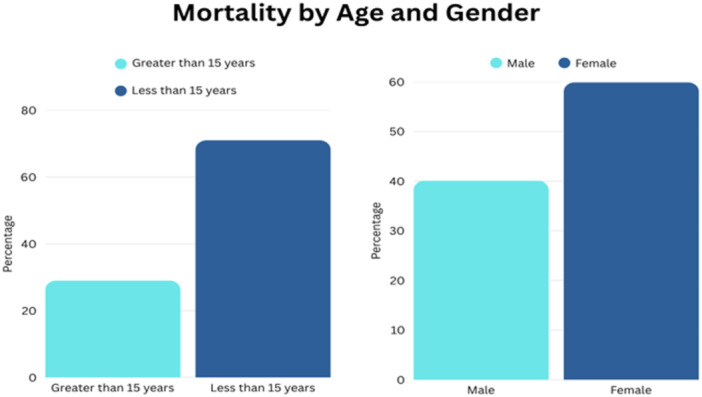
Distribution of patients by their respective percentage mortality.

Children ages 0–14 make up 64.3% of the instances recorded in the Panzi health region. Age ranges of 0–59 months make up 53%, the 5–9 years age bracket accounts for 7.4%, and 10–14 years have 3.9% of the total cases, respectively. Of the overall reported incidents, 59.9% are female. Of the deaths, 71% occur in people under the age of 15, with children under the age of five accounting for 54.8% of the total fatalities. Nine of the 145 patients who were 15 years of age or older passed away. Malnutrition was identified in all severe incidents. It was noted that the majority of the fatalities had taken place in the local settlements [1]. Given that malaria, respiratory viral infections, anemia, and malnutrition are endemic in the region, their detection in preliminary investigations should be interpreted cautiously, and the possibility of additional etiological factors—including arboviral infections—remains under investigation. Figure 1 shows the distribution of patients by their respective percentage mortality.

## 5 Clinical Presentation and Risk Factors of Disease X

**FIGURE 2:**
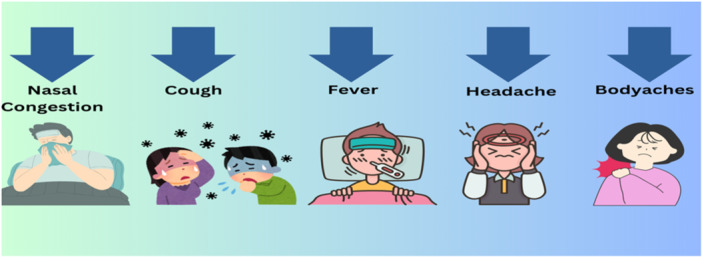
Different symptoms observed in Disease X.

According to the early reports, Remy Saki, the deputy governor of Kwango province, people with this unknown disease experienced symptoms similar to the flu, such as a fever and intense headaches [3]. WHO reported that body aches, headaches, coughs, nasal congestion, and fever were the major symptoms [1]. These commonly presented symptoms are illustrated in Figure 2. Individuals present clinically with symptoms of fever in 96.5% of the cases, cough in 87.9% of the affected, exhaustion and fatigue in 60.9%, and runny nose and congestion in 57.8% of the patients, respectively [1]. Of the total 31 deaths, problems with breathing, anemia, and severe malnutrition were the primary symptoms [1].

According to WHO, malnutrition was found in all the severe cases. Children, especially those under the age of five, account for the greatest number of instances that were recorded. The regions that were isolated and rural and the ones affected by the wet season, which made access even more difficult, were the most vulnerable to this unknown disease. Most of the deaths have taken place in the local rural settlements. In addition to low immunization rates, the region has seen a significant worsening of food insecurity and has very limited access to high‐quality case care and testing. In addition to a shortage of medical personnel, the area lacked supplies and transportation options [1]. In DRC, according to the Integrated Food Security Phase Classification (IPC), nearly 4.5 million children between the ages of 6 and 59 months are experiencing or are predicted to experience acute malnutrition between July 2024 and June 2025, with around 1.4 million instances of serious acute malnutrition and 3.1 million instances of moderate acute malnutrition. Additionally, 3.7 million pregnant and lactating women are anticipated to experience or experiencing acute malnutrition during the same time frame [8]. So, it can be concluded that inaccessible rural areas, malnutrition, young age, and lack of health resources are among the major risk factors for this recent outbreak.

## 6 Historical Perspective of the Previous Outbreaks

The vast region stretching the Intertropical Convergence Zone comprises a wide range of nations with a range of plant life, from rainforest to somewhat barren [9]. Throughout the 21st century, numerous disease epidemics have appeared or resurfaced throughout Africa. While some of these are recognized to be ancient illnesses like cholera and plague, others are connected to recently identified infections or illnesses. Furthermore, conditions linked to previously identified microbes, such as the Zika and Ebola viruses, have lately been linked for the first time to widespread outbreaks with worldwide repercussions [10]. A quarter of all deaths worldwide at the start of the 21st century were caused by disease epidemics, which killed more than 10 million individuals annually, mostly in low and middle‐income countries. New infectious illnesses affect the worldwide economy and present a serious public health concern. Its origin typically depends on economic and social circumstances, as well as environmental variables. Biodiversity in poor nations in tropical Africa is mostly at risk for zoonotic and vector‐borne infectious diseases. In all, zoonotic diseases account for 60% of newly discovered disease outbreaks, with animal‐borne illnesses accounting for 72% of these cases [11, 12]. For example, in the case of malaria, in 2022, there were a total of 249 million instances of malaria globally, and 608,000 deaths were projected. The WHO concluded that children under the age of five contribute to 78% of deaths caused by malaria in the African area, which bears a significant burden, accounting for about 94% of incidence and 95% of mortality in 2022. Particularly, almost half of the malaria‐related fatalities worldwide occurred in the Democratic Republic of the Congo, Nigeria, Uganda, and Mozambique combined [13]. Yellow fever, which is indigenous to tropical and subtropical parts of Africa and the South American continent, is spread by mosquito bites, just like malaria. In Africa, there are between 84,000 and 170,000 serious cases of yellow fever and up to 60,000 fatalities [14]. Cholera is a concerning global medical issue that affects about 2.9 million individuals each year and kills 95,000 people worldwide, most of whom live in low‐ to middle‐income nations [15]. Over 40 million individuals reside in cholera‐affected regions across Africa alone. Since January 1, 2022, the WHO Regional Office for Africa (AFRO) has received reports of over 250,000 cholera cases, including 4695 fatalities [15]. In addition, in any particular year, Africa is responsible for 25% of all tuberculosis(TB) cases around the world, which translates to 417,000 deaths out of the almost two million TB‐related deaths that occur globally [16]. There were 118 outbreaks reported in the WHO African area in 2021, up from 106 in 2020. One hundred twenty‐nine outbreaks, many of which crossed borders, have been reported in the same area by October 2022. Between 2001 and 2022, there were 1800 public health emergencies (PHEs) in the African continent, most of which were related to newly developing infectious diseases [17]. These examples above emphasize the recurring challenges and impact of different infectious diseases across the African continent.

## 7 Key Challenges

### 7.1 Emerging Pathogens and Drug Resistance

One persistent problem is the unpredictable and persistent nature of infectious illness onset. Infectious illnesses still claim millions of lives every year despite enormous advancements, especially in the previous 20 years. More virulent pathogens keep emerging and resurfacing. For African nations, managing the multifaceted nature of new and re‐emerging infectious diseases is an ongoing challenge [12]. Because dangerous microbes are able to adapt to new or preexisting hosts, genetic changes in these microbes present a significant issue. Resistance to existing medications can lead to increased mortality. Antiretroviral therapy effectiveness is decreased, and regimen modifications are required due to high genomic diversity during virus replication and drug pressure [18].

### 7.2 Healthcare Related Challenges

Medical facilities in sub‐Saharan Africa deal with problems like a rise in non‐communicable diseases, inadequate monitoring of illnesses, conflicts, and insufficient investment [19]. Critical limitations of diagnostic systems, protective gear for individuals, and healthcare personnel make vigilant monitoring problematic. The lack of digital archives results in poorer data quality. African states struggle with violence, volatile politics, insufficient infrastructure, and poor administration [20]. Poor monitoring of diseases is linked to high workloads for staff, fatigue and lack of motivation among healthcare individuals, and an overall shortage of qualified healthcare personnel. Cross‐infections arise from inadequate equipment and isolation rooms, which worsen infection management. The lack of diagnostic capabilities in susceptible populations also increases the transmission of these emerging new infections [18].

### 7.3 Socio‐Economic Challenges

Among the prevalent issues include low socioeconomic conditions, wide‐ranging poverty, and restricted access to medical care. In Sub‐Saharan Africa, where 34% of families live in severe poverty, a number of diseases are caused by overcrowding and poor access to water, sanitation and hygiene. Twenty percent of Africans suffer from malnutrition, which increases vulnerability and reduces available treatments [20]. Infectious disease transmission is accelerated by a lack of health education and low awareness of risks, which result in less knowledge, fewer vaccinations, and delayed treatment [18]. Illegal border crossing makes it easier to spread infectious diseases and makes tracking and tracing of individuals more difficult in these African countries. Social stigma has prevented people from obtaining healthcare, and hostility towards the governments and healthcare institutions has resulted in reduced rates of vaccination and treatment, as observed in areas affected by a number of infectious illnesses [21]. Finally, old customs have also contributed to the spread of these diseases, for example, in West Africa, customary burial practices like bathing corpses have contributed to the spread of diseases like Ebola [22].

## 8 Global Health Security and Response

To raise awareness of the significance of the One Health concept worldwide, WHO is emphasizing the “One Health Day” campaign. It focuses on understanding how human behavior and policy may impact the health of animals and the environment. In order to prevent zoonotic and vector‐borne diseases from reoccurring, it focuses on ensuring food safety, decreasing antimicrobial‐resistant infections, and addressing environmental issues [23]. An approach for creating and executing effective public health monitoring and management systems in African nations was adopted in 1998 by the WHO's Regional Office for Africa (AFRO) and its collaborators. These focused on grassroots efforts in surveillance and early identification of diseases, quick confirmation, and reaction to public health hazards in order to combat the newly emerging global infection‐related risks [24]. The US government, with the help of USAID, has worked in countries like the DRC to focus on interconnected threats like antibiotic resistance and emerging animal diseases like Ebola and other novel viruses. It primarily focuses on developing long‐term infectious disease capabilities by enhancing disease surveillance and detection through laboratory systems. It also works on increasing the nation's ability to identify and describe new viruses that emerge from wildlife, comprehend how these viruses could infect people, and enhance readiness to stop, identify, and effectively handle pandemic risks [25].

## 9 Current Response to Disease X

### 9.1 Coordination

At the local and national levels, coordination has improved. A rapid response team (RRT) from Kwango Province was sent to Panzi following the initial Public Health Emergency Operations Center (PHEOC) meeting with all partners on November 30, 2024, to discuss the alarming situation. Provincial teams are actively involved in continuing planning; in addition, daily meetings are held at the higher level to address the ongoing situation (1).

### 9.2 Surveillance and Management

Currently, medical facilities are actively searching for cases, by reviewing hospital records to find more cases. In the wider community, aggressive case searches and probes are also being planned. In order to gain a better understanding of the factors that contribute to the disease spread and the extent of the outbreak, community fatalities are also being examined [1]. To look into the epidemic and strengthen the response, a provincial Rapid Response Team was sent to Panzi on November 30, 2024, and a multidisciplinary team comprising WHO experts was sent to the region on December 7, 2024 [1].

### 9.3 Laboratory and Logistics

In order to gather samples from patients and transfer them to the National Institute of Biomedical Research (INRB) in Kinshasa for analysis, lab equipment was provided. Rapid diagnostic tests for COVID‐19 and malaria have also been made available to aid in diagnosis. Samples are being transported to INRB Kinshasa for laboratory testing as part of the logistical support offered for the efficient handling of cases. To aid in the recovery process, hospitals and other medical facilities in the most impacted areas are receiving the necessary drugs and sample kits [1].

### 9.4 Prevention of Infection and Communication

Healthcare workers have been informed on key methods, such as proper application of masks, hand washing, and gloves to minimize the possibility of further dissemination, and important instructions have been developed to increase public understanding and promote general precautions. These recommendations are being spread through outreach to the public, with education efforts underway. These measures to prevent and control this infection are being reinforced [1].

## 10 Future Recommendations

### 10.1 Public Health Education

In order to mitigate and control developing illnesses in Africa, public health education is essential. It provides the general public and healthcare professionals with the information base upon which to build decisions in the areas of preventing illness and health improvement [26]. In order to create a population that is less susceptible to newly emerging and infectious illnesses, this active strategy of health awareness focuses on encouraging individuals to practice a healthy approach while opposing behaviors that may lead to infection [27]. According to WHO, for a cohesive response against disease X, coordinating mechanisms spanning all levels must be strengthened. To get around the restricted network coverage in impacted locations, improved communication infrastructure—like satellite phones is needed. To gain a better understanding of disease patterns and mortality, it is imperative to improve surveillance operations. Health facilities and communities should continue to conduct active case searches, paying special attention to family clusters and regions where deaths have been reported. To guarantee early case discovery and prompt intervention, community‐based tracking and surveillance need to be reinforced [2].

### 10.2 Active Research and Vaccination

A diversified approach is needed to efficiently combat infectious illnesses that are resurfacing and developing. Persistent research and development of broadly protective immunizations employing innovative technology are essential to keep up with developing infections [28]. Identifying all possible situations and prioritizing widespread vaccinations to avoid serious outbreaks, interrupt viral replication, and guard against future varieties are essential [29]. All these challenges are difficult to counter, considering the socio‐ economic landscape of DRC [20].

### 10.3 Early Detection and Surveillance

Prioritizing and enhancing surveillance and response capacities is essential. Although there has been progress in lowering the prevalence of tropical diseases, communities with few resources still struggle to create less conducive environments for pathogens [30]. The necessity of incorporating surveillance data into focused, prompt public health interventions is shown by the worldwide collaboration exhibited by smallpox and polio eradication initiatives. Information from efficient monitoring systems is essential for the prevention, management, and eradication of illness [31].

### 10.4 Global Systematic Approach

**FIGURE 3:**
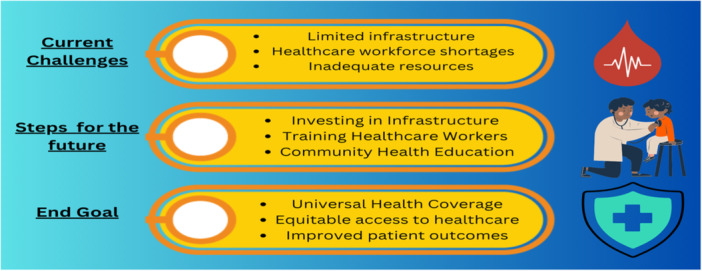
Pathway to strengthening healthcare system.

A One Health system is necessary to enhance cooperation and coordination in the fight against newly developing and reappearing infectious diseases throughout Africa [32]. In order to avoid, detect, and react to emerging zoonotic diseases, a One Health approach that incorporates cooperative efforts in program development, drafting policies, regulation, and research works well. A coordinated approach can improve medical outcomes for people in Africa and globally while producing affordable solutions with limited funds [33, 34]. A summary of the challenges, their response, and the end goal is shown in Figure 3.

### 10.5 Improving Infrastructure and Universal Health Coverage

Medical facilities must be strengthened by making investments in robust infrastructure, educating employees, and promoting community involvement. Reducing epidemic damage requires preventive planning, which involves governance frameworks, adaptable procedures, and frequent drills [35]. For Africa to be ready for new and reemerging infectious illnesses in the future, Universal Health Coverage (UHC) must be prioritized. It ensures that everyone has an equal opportunity to receive quality treatment without facing financial strain [36]. Adopting UHC not only offers medical and economic advantages but also advances social equality by lessening the load on the poor. The financial impact of the epidemic of Ebola serves as evidence that UHC can prevent financial disasters, encourage early identification of illness, and protect against economic crises [36, 37]. WHO also recommends to promote treatment and stop additional fatalities, effective case management necessitates making sure that there is a sufficient supply of necessary medications and oxygen therapy is accessible, and that healthcare personnel are trained in basic emergency and critical care. Distribution of rapid kits for malaria is necessary to enable early diagnosis and timely treatment. Decentralization and long‐term laboratory capacity building will be crucial for early death cause detection and diagnostic capacity providing in the impacted health zone [2]. Similar strategies can be implemented for future outbreaks.

## 11 Conclusion

The outbreak of the unidentified disease in the DRC shows a pressing need to strengthen healthcare infrastructure, employ better diagnostic techniques, and devise improved response schemes and strategies. Addressing issues like malnutrition and the lack of healthcare facilities is essential for treating the current crisis and preventing any future outbreaks. The affected people can significantly benefit by strengthening the healthcare system with local and international efforts and support. Further investigations led by Congolese research teams, in collaboration with international public health partners, are essential to accurately determine the etiological factors responsible for the outbreak and to strengthen coordinated response strategies.

## Author Contributions

**Muhammad Shaheer Bin Faheem:** conceptualization, writing – original draft, writing – review and editing, visualization, validation, methodology, project administration, supervision, data curation. **Muhammad Haroon‐Ul‐Rasheed:** writing – original draft, methodology, visualization, resources. **Sumaya Samadi:** writing – review and editing, resources, methodology.

## Funding

The authors have nothing to report.

## Conflicts of Interest

The authors declare no conflicts of interest.

## Transparency Statement

The lead author, Sumaya Samadi, affirms that this manuscript is an honest, accurate, and transparent account of the study being reported; that no important aspects of the study have been omitted; and that any discrepancies from the study as planned (and, if relevant, registered) have been explained.

## Data Availability

The authors have nothing to report.
