# Correction to “Mystery Disease X Outbreak in the Democratic Republic of the Congo: A Narrative Review of Epidemiological Patterns and Response Challenges”

**DOI:** 10.1002/hsr2.72540

**Published:** 2026-05-17

1

M. S. B. Faheem, M. Haroon‐Ul‐Rasheed, and S. Samadi, “Mystery Disease X Outbreak in the Democratic Republic of the Congo: A Narrative Review of Epidemiological Patterns and Response Challenges,” Health Science Reports9 (2026): e72263, https://doi.org/10.1002/hsr2.72263.

In the Transparency Statement section, the lead author's name was incorrect. The section should read:

Transparency Statement

The lead author, Muhammad Shaheer Bin Faheem, affirms that this manuscript is an honest, accurate, and transparent account of the study being reported; that no important aspects of the study have been omitted; and that any discrepancies from the study as planned (and, if relevant, registered) have been explained.

The online version of this article has been corrected accordingly.

We apologize for this error.

